# Myasthenia Gravis in Pregnancy: A Case Report

**DOI:** 10.1155/2012/736024

**Published:** 2012-01-26

**Authors:** Sebastian Berlit, Benjamin Tuschy, Saskia Spaich, Marc Sütterlin, Regine Schaffelder

**Affiliations:** Department of Obstetrics and Gynecology, University Medical Center Mannheim, 68167 Mannheim, Germany

## Abstract

*Objective*. To present a case of maternal myasthenia gravis in pregnancy and give a systematic review of the literature. *Case*. We report the case of a 38-year-old parturient with a life-threatening complication of immune-mediated myasthenia gravis shortly after an elective cesarean section on patient's request under spinal anesthesia at 35 + 3 weeks of gestation. The newborn was transferred to the pediatric unit for surveillance and did not show any signs of muscular weakness in the course of time. The mother developed a respiratory insufficiency on the second day postpartum. The myasthenic crisis led to a progressive dyspnoea within minutes, which exacerbated in a secondary generalized seizure with cardiac-circulatory arrest. After successful cardiopulmonary resuscitation, the patient was transferred to intensive care. The interdisciplinary therapeutic approach included ventilatory assistance via endotracheal intubation, parenteral pyridostigmine, azathioprine, and steroids. By interdisciplinary measures, a stable state was regained. *Conclusion*. Myasthenia gravis especially when associated with pregnancy is a high-risk disease. As this disease predominantly occurs in women of reproductive age, it is important to be aware of this condition in obstetrics and its interdisciplinary diagnostic and therapeutic management.

## 1. Introduction

The heterogeneous group of congenital and acquired myasthenia gravis (MG) syndromes is clinically characterized by an insufficient neuromuscular transmission leading to progressive paresis. Myasthenia gravis is an autoimmune disorder of the neuromuscular transmission, caused by autoantibodies against the nicotinic acetylcholine receptor, hence leading to an insufficient nerve impulse transmission to striated muscle fibers [1]. These antibodies of the IgG isotype are detected in 80–90% of generalized MG and in 50–70% of ocular MG [2]. Seronegative MG is caused by humoral factors. In 40% of patients with seronegative MG IgG antibodies against the muscle specific kinase (MuSK) are found and do not occur in patients with seropositive MG [3, 4].

Prevalence of MG lays between 1 in 10.000 and 1 in 50.000, with 2/3 of affected individuals being female. Commonly women in their second and third decades of life, hence their reproductive years, are affected [5, 6].

In the case of maternal myasthenia gravis, both the mother and the child may develop myasthenia symptoms with varying degrees of weakness and progressive fatigability of the skeletal muscles. Therefore, in this paper, we attempt to summarize inevitable interdisciplinary diagnostic and therapeutic strategies considering the medical management in pregnancy as well as the puerperal and neonate period by a systematic literature review.

Data for the case report were generated by reviewing labour, delivery, and postpartal records.

The database of the US National Library of Medicine (PUBMED) was used to identify publications being published from 1966 to June 2011.

## 2. Case Report

We report the case of a 38-year-old, previously thymectomized patient with immune-mediated MG, who underwent an elective cesarean operation under spinal anesthesia at 35 + 3 weeks of pregnancy, after having had a premature rupture of membranes. Upon admission, the patient had no contractions and showed no signs of muscular weakness perceiving a sufficient medication (pyridostigmine bromide, prednisone, azathioprine). In the course of pregnancy, there was an initial progress of myasthenic symptoms, which disappeared completely after adjusting medication as stated above. Elective cesarean section was performed on patient's request. The surgery was implemented without complications. A lively male infant (weight: 3120 g, length: 51 cm, head circumference: 35 cm; APGAR score: 9/10/10) was born and did not show any signs of muscular weakness.

The newborn was transferred to the pediatric unit for surveillance and did not show any signs of neonatal MG initially as well as in the course of time.

The mother developed a respiratory insufficiency on the second postpartal day. The myasthenic crisis led to progressive dyspnoea which exacerbated in a secondary generalized seizure with cardiac-circulatory arrest. After successful cardiopulmonary resuscitation, the patient was transferred for intensive care treatment.

Blood values showed an elevated antibody titer (Ach-R-Ak 54.5 nmol/L, normal range <0.4 nmol/L) as well as slightly elevated inflammation values (CRP 15 mg/dL, normal range <5 mg/dL; leucocytes 12.000/*μ*L, normal range 4.000–9.400/*μ*L), a computed tomography did not show any cerebral pathologies, but a lobar pneumonia was detected.

The interdisciplinary therapeutic approach included ventilatory assistance via endotracheal intubation, parenteral antibiotics (piperacillin and tazobactam), pyridostigmine, azathioprine, and corticosteroids. By these contemplated measures, a stable state was regained, so that after five days of intensive care treatment the patient was transferred to normal ward. On the eleventh postpartal day, the patient could be dismissed in good clinical condition.

## 3. Discussion

The course of MG in pregnancy as well as its influence on pregnancy outcome is unpredictable. It has been shown that in 31% of patients the disease remained stable during pregnancy, whereas in 29% an improvement and in 40% an exacerbation of myasthenia symptoms were observed [5]. Generally worsening of clinical symptoms occurs in about one-third of MG patients. Although possible at any state during pregnancy, it is more likely during the first trimester and the first month postpartum [7–9]. An improvement of symptoms has been observed in 20–40% of patients in the second and third trimesters of pregnancy [7]. The clinical course of MG throughout the first pregnancy does not predict the clinical course of subsequent pregnancies [9].

Reviewing the literature, there is no great influence of MG on pregnancy.

The incidence of spontaneous abortion and growth restriction is not increased [9, 10]. MG does not increase the incidence of prematurity, but frequency of premature rupture of membranes, as stated in the case above, is increased [10]. Preterm delivery rate in patients with MG is associated with an increased incidence between 13 and 41.3% [5, 11].

There is no increase of incidence of preeclampsia in pregnant women with MG, although some cases have been described [12, 13]. Noteworthy is the fact that the use of magnesium sulfate is contraindicated in patients with MG [13].

Generally patients with MG should be observed closely throughout pregnancy by a neurologist and an obstetrician. Some authors suggest that there should be regular ultrasound checkups in order to detect fetal akinesia, manifesting itself by a reduction of fetal movements and breathing motion, as well as hydramnios [14].

Generally hypoventilation in MG patients is a risk. Pregnancy leads to an aggravation of respiratory complications, as movement of the diaphragm is limited due to an enlargement of the uterus. A respiratory crisis as described in our case is one of the most severe complications. An immediate interdisciplinary therapeutic approach is necessary.

MG maternal mortality risk is inversely correlated to the duration of the disease, the highest in the first year and the lowest risk 7 years after the beginning of the disease [15]. Pregnancy does not worsen the long-term outcome of MG [9, 10].

Several therapeutic strategies and approaches have to be taken into account in patients with MG. Generally patients with MG should be educated to avoid unnecessary physical activities to diminish fatigue. Before planning pregnancy, there should be an informed decision regarding the medical management of MG throughout pregnancy explaining fetal and maternal risks. Medical advice should be based upon severity of MG [16, 17].

Thymectomy has been recommended for the treatment of MG and is a primary disease controlling modality. The incidence of clinical exacerbations seems to be higher in nonthymectomized than thymectomized patients, while there is no difference in the development of neonatal MG between the two groups [11]. Still, complete remission of the disease has been described in approximately 45% of thymectomized patients and clinical improvement is not noted until years after surgery [18, 19]. Therefore, the authors state that an operative therapy can be postponed until after delivery, since a delay does not adversely affect patient's outcome.

Medical treatment should not be altered in pregnancy. Anticholinesterase inhibitors have been safely used in pregnant patients with MG, and in 50% of patients monotherapy is sufficient [7]. Only few cases of fetal malformations such as severe neonatal MG with microcephaly have been described [20].

Corticosteroid therapy appears to be safe during pregnancy with little teratogenic risk [21–23]. Only a slight increase in incidence of cleft palate has been described [24]. High-dose corticosteroid therapy increases the risk of premature rupture of membranes [25].

Azathioprine is not recommended in pregnancy as fetuses exposed have an increased risk of myelosuppression [26, 27].

As some previous publications show, cyclosporine A is not necessarily associated with severe effects to the fetus, although an increase of risk for fetuses small for gestational age, fetal myelosuppression, prematurity, and spontaneous abortion have been observed [28, 29]. Mycophenolate mofetil [30], methotrexate [31], and cyclophosphamide [32, 33] are contraindicated in pregnancy. Plasmapheresis on the other hand has been safely accomplished throughout pregnancy especially when a short-time benefit is needed [7, 9]. Due to the removal of hormones via plasmapheresis, the risk for prematurity is increased [34]. The use of intravenous immunoglobulins in pregnancy is still experimental but seems to be an effective and safe therapeutic approach [35].

Regarding the mode of delivery, a vaginal birth should be preferred as the uterus is not affected by autoantibodies as it does not consist of striated muscle [7, 17]. As striated muscle is involved in the second stage of labour, a vacuum extraction or forceps delivery might be necessary if obstetrical assistance is required. Cesarean section should only be performed if there is an obstetric indication [7]. Generally epidural anesthesia should be preferred and narcotic analgesia and muscle relaxants avoided [36].

Neonatal MG has been observed in 10–20% of newborns of parturients with MG [37, 38]. Neuromuscular symptoms in newborns manifest clinically within the first 12–48 hours postpartum, so that a rigorous monitoring is inevitable [39]. In general there is no correlation between the severty of maternal MG and the occurrence of a neonatal MG [9, 37, 40]. Furthermore cases of pulmonary hypoplasia, arthrogryposis congenital, and nonspecific hyperbilirubinemia have been reported [7, 41, 42]. These fetal complications rarely occur. MG patients should still be informed that, although the clinical state throughout pregnancy concerning MG is stable, fetal complications might occur.

Breastfeeding is not contraindicated in parturients with MG, although serum antibodies versus acetylcholine receptors might reach the newborn via breast milk, so that neonatal MG might be enhanced [43]. A nonsignificant transfer of pyridostigmine bromide and corticosteroids has been shown in various publications [43, 44]. Authors suggest to defer breastfeeding until 4 hours after having taken the above-mentioned medication [45]. Breastfeeding is contraindicated in mothers taking mycophenolate mofetil, cyclophosphamide, methotrexate, azathioprine, cyclosporine A [44].

## 4. Conclusion

Myasthenia gravis especially when associated with pregnancy is a high-risk disease, and its course is unpredictable. Severe up to life-threatening conditions might occur especially due to generalized weakness, in particular respiratory insufficiency endangering the parturient as well as the newborn. As this disease predominantly occurs in women of reproductive age, it is important to be aware of this condition and its interdisciplinary diagnostic and therapeutic management.

## References

[1] Tzartos SJ, Barkas T, Cung MT (1998). Anatomy of the antigenic structure of a large membrane autoantigen, the muscle-type nicotinic acetylcholine receptor. *Immunological Reviews*.

[2] Lindstrom J (1977). An assay for antibodies to human acetylcholine receptor in serum from patients with myasthenia gravis. *Clinical Immunology and Immunopathology*.

[3] Vincent A, Bowen J, Newsom-Davis J, McConville J (2003). Seronegative generalised myasthenia gravis: clinical features, antibodies, and their targets. *Lancet Neurology*.

[4] Vincent A, McConville J, Farrugia ME, Newsom-Davis J (2004). Seronegative myasthenia gravis. *Seminars in Neurology*.

[5] Plauche WC (1983). Myasthenia gravis. *Clinical Obstetrics and Gynecology*.

[6] Kurtzke JF (1978). Epidemiology of myasthenia gravis. *Advances in Neurology*.

[7] Djelmis J, Sostarko M, Mayer D, Ivanisevic M (2002). Myasthenia gravis in pregnancy: report on 69 cases. *European Journal of Obstetrics Gynecology and Reproductive Biology*.

[8] Plauche WC (1991). Myasthenia gravis in mothers and their newborns. *Clinical Obstetrics and Gynecology*.

[9] Batocchi AP, Majolini L, Evoli A, Lino MM, Minisci C, Tonali P (1999). Course and treatment of myasthenia gravis during pregnancy. *Neurology*.

[10] Hoff JM, Daltveit AK, Gilhus NE (2003). Myasthenia gravis: consequences for pregnancy, delivery, and the newborn. *Neurology*.

[11] Eden RD, Gall SA (1983). Myasthenia gravis and pregnancy: a reappraisal of thymectomy. *Obstetrics and Gynecology*.

[12] Duff GB (1979). Preeclampsia and the patient with myasthenia gravis. *Obstetrics and Gynecology*.

[13] Cohen BA, London RS, Goldstein PJ (1976). Myasthenia gravis and preeclampsia. *Obstetrics and Gynecology*.

[14] Stoll C, Ehret-Mentre MC, Treisser A, Tranchant C (1991). Prenatal diagnosis of congenital myasthenia with arthrogryposis in a myasthenic mother. *Prenatal Diagnosis*.

[15] Scott JS (1977). Immunological diseases in pregnancy. *Progress in Allergy*.

[16] Igarashi S, Yamauchi T, Tsuji S, Furukawa T, Tanoue K, Miyatake T (1992). A case of myasthenia gravis complicated by cyclic thrombocytopenia. *Clinical Neurology*.

[17] Klehmet J, Dudenhausen J, Meisel A (2010). Course and treatment of myasthenia gravis during pregnancy. *Nervenarzt*.

[18] Rowland JM, Hendrickx AG (1983). Corticosteroid teratogenicity. *Advances in Veterinary Science and Comparative Medicine*.

[19] Bever CT, Aquino AV, Penn AS (1983). Prognosis of ocular myasthenia. *Annals of Neurology*.

[20] Niesen CE, Shah NS (2000). Pyridostigmine-induced microcephaly. *Neurology*.

[21] Fraser FC, Sajoo A (1995). Teratogenic potential of corticosteroids in humans. *Teratology*.

[22] Martínez-Rueda JO, Arce-Salinas CA, Kraus A, Alcocer-Varela J, Alarcón-Segovia D (1996). Factors associated with fetal losses in severe systemic lupus erythematosus. *Lupus*.

[23] Czeizel AE, Rockenbauer M (1997). Population-based case-control study of teratogenic potential of corticosteroids. *Teratology*.

[24] Rodríguez-Pinilla E, Martínez-Frías ML (1998). Corticosteroids during pregnancy and oral clefts: a case-control study. *Teratology*.

[25] Gaudier FL, Santiago-Delpin E, Rivera J, Gonzales Z (1988). Pregnancy after renal transplantation. *Surgery Gynecology and Obstetrics*.

[26] DeWitte DB, Buick MK, Cyran SE, Maisels MJ (1984). Neonatal pancytopenia and severe combined immunodeficiency associated with antenatal administration of azathioprine and prednisone. *Journal of Pediatrics*.

[27] Davison JM, Dellagrammatikas H, Parkin JM (1985). Maternal azathioprine therapy and depressed haemopoiesis in the babies of renal allograft patients. *British Journal of Obstetrics and Gynaecology*.

[28] Radomski JS, Ahlswede BA, Jarrell BE (1995). Outcomes of 500 pregnancies in 335 female kidney, liver, and heart transplant recipients. *Transplantation Proceedings*.

[29] Bermas BL, Hill JA (1995). Effects of immunosuppressive drugs during pregnancy. *Arthritis and Rheumatism*.

[30] Armenti VT, Radomski JS, Moritz MJ (2002). Report from the National Transplantation Pregnancy Registry (NTPR): outcomes of pregnancy after transplantation. *Clinical Transplants*.

[31] Buckley LM, Bullaboy CA, Leichtman L, Marquez M (1997). Multiple congenital anomalies associated with weekly low-dose methotrexate treatment of the mother. *Arthritis and Rheumatism*.

[32] Blatt J, Mulvihill JJ, Ziegler JL (1980). Pregnancy outcome following cancer chemotherapy. *American Journal of Medicine*.

[33] Murray CL, Reichert JA, Anderson J, Twiggs LB (1984). Multimodal cancer therapy for breast cancer in the first trimester of pregnancy. A case report. *Journal of the American Medical Association*.

[34] Samuels P, Pfeifer SM (1989). Autoimmune diseases in pregnancy. The obstetrician’s view. *Rheumatic Disease Clinics of North America*.

[35] Ellison J, Thomson AJ, Walker ID, Greer IA (2000). Thrombocytopenia and leucopenia precipitated by pregnancy in a woman with myasthenia gravis. *British Journal of Obstetrics and Gynaecology*.

[36] Astley BA, Hughes R, Payne JP (1987). Recovery of respiration after neuromuscular blockade with alcuronium. *British Journal of Anaesthesia*.

[37] Donaldson JO, Penn AS, Lisak RP (1981). Antiacetylcholine receptor antibody in neonatal myasthenia gravis. *American Journal of Diseases of Children*.

[38] Bartoccioni E, Evoli A, Casali C (1986). Neonatal myasthenia gravis: clinical and immunological study of seven mothers and their newborn infants. *Journal of Neuroimmunology*.

[39] Papazian O (1992). Transient neonatal myasthenia gravis. *Journal of Child Neurology*.

[40] Lefvert AK, Osterman PO (1983). Newborn infants to myasthenic mothers: a clinical study and an investigation of acetylcholine receptor antibodies in 17 children. *Neurology*.

[41] Vincent A, Newland C, Brueton L (1995). Arthrogryposis multiplex congenita with maternal autoantibodies specific for a fetal antigen. *Lancet*.

[42] Daskalakis GJ, Papageorgiou IS, Petrogiannis ND, Antsaklis AJ, Michalas SK (2000). Myasthenia gravis and pregnancy. *European Journal of Obstetrics Gynecology and Reproductive Biology*.

[43] Brenner T, Shahin R, Steiner I, Abramsky O (1992). Presence of anti-acetylcholine receptor antibodies in human milk: possible correlation with neonatal myasthenia gravis. *Autoimmunity*.

[44] Ward RM, Bates BA, Benitz WE (2001). The transfer of drugs and other chemicals into human milk. *Pediatrics*.

[45] Burakoff R, Opper F (1995). Pregnancy and nursing. *Gastroenterology Clinics of North America*.

